# Protective effects of *Angelica sinensis* polysaccharide on chemotherapy-injured rats with premature ovarian insufficiency and its impact on gut microbiota

**DOI:** 10.3389/fphar.2025.1685281

**Published:** 2025-11-19

**Authors:** Maoyuan Wu, Haixia Shu, Min Wang, Yan Xu, Yunhan Zhang, Xiaoling Jiang, Lixia Zhang, Xuemei Chen, Lianli He

**Affiliations:** Department of Gynecology and Obstetrics, The Third Affiliated Hospital of Zunyi Medical University (The First People’s Hospital of Zunyi), Zunyi, Guizhou, China

**Keywords:** premature ovarian insufficiency, *Angelica sinensis* polysaccharide, oxidative stress, gut microbiota, chemotherapy

## Abstract

**Objective:**

This study aimed to investigate the therapeutic effects and action mechanisms of *Angelica sinensis* polysaccharides (ASP) on a chemotherapy-induced premature ovarian insufficiency (POI) rat model along with screening for the optimal therapeutic dose.

**Methods:**

Sprague–Dawley female rats were used to establish a POI rat model via intraperitoneal injection of cyclophosphamide and busulfan. The rats were treated with ASP at doses of 80, 160, and 320 mg/kg/d for 21 d. The ovarian histomorphology and follicular development were examined by hematoxylin and eosin staining, and the serum levels of follicle-stimulating hormone (FSH), luteinizing hormone (LH), estradiol (E2), anti-Müllerian hormone (AMH), and inflammatory cytokines (IL-6, IL-1β, and TNF-α) were measured. The ovarian oxidative stress was assessed via malondialdehyde (MDA), superoxide dismutase (SOD), glutathione peroxidase (GSH-Px), and reactive oxygen species (ROS) levels. The composition of the gut microbiota was analyzed by 16S rDNA sequencing, and the associations between differential microbiota and ovarian indicators were assessed using Spearman correlation analysis.

**Results:**

ASP treatment improved sex hormone secretion in the chemotherapy-induced POI rats, in addition to increased E2 and AMH levels, decreased FSH and LH levels, improved ovarian tissue structure, and increased follicle growth at all stages. ASP treatment also improved the serum inflammatory levels in POI rats by reducing the IL-6, IL-1β, and TNF-α levels; it decreased oxidative stress levels in the ovarian tissue, inhibited ROS and MDA activities, and increased SOD and GSH-Px activities. The gut microbiota differential analysis showed that chemotherapy-induced POI rats exhibited dysbiosis of the gut microbiota. After ASP treatment, the α and β diversities of the gut microbiota changed, thereby increasing the relative abundance of beneficial bacteria and decreasing the relative abundance of harmful bacteria. Spearman’s correlation analysis was performed between the main differential microbiota as well as serum sex hormone, proinflammatory cytokine, and ovarian tissue oxidative stress levels; accordingly, the results showed that some beneficial bacteria were positively correlated with E2, AMH, SOD, and GSH-Px levels while being negatively correlated with FSH, LH, MDA, IL-6, IL-1β, and TNF-α levels.

**Conclusion:**

ASP ameliorates chemotherapy-induced POI in rats by improving the serum hormone levels, promoting follicular development, as well as suppressing inflammation and oxidative stress, with medium and high treatment doses showing significant efficacies. Furthermore, ASP reshapes the gut microbiome; the altered gut microbiota are strongly correlated with ovarian function indicators, suggesting that they may serve as a new therapeutic approach for POI.

## Graphical Abstract

**Figure:**
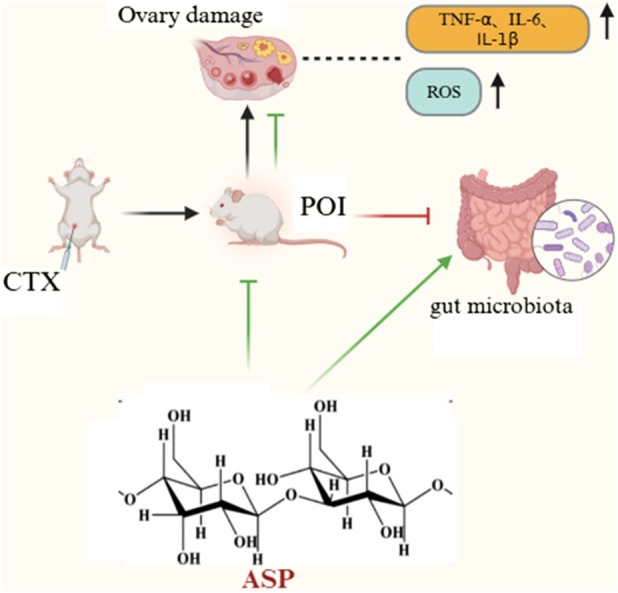
The graphical abstract demonstrates the improvement of ovarian function by *Angelica sinensis* polysaccharide against chemotherapy-induced premature ovarian insufficiency in rats.

## 1 Introduction

Premature ovarian insufficiency (POI) is a common gynecological endocrine disorder that refers to the condition in which female patients experience a decline in ovarian function before the age of 40 years. The main manifestations of POI include scanty menstruation or even amenorrhea, elevated follicle-stimulating hormone (FSH) level (FSH > 25 U/L), and reduced estrogen level (Panay et al., 2024). POI seriously affects the physical and mental health as well as fertility of women. Its etiology is mainly related to autoimmunity, genetic defects, gene mutations, medical factors, and environmental factors (Bunpei, 2021). With the “rejuvenation” of tumors, chemotherapy-induced or medically induced POIs can impede the reproductive needs of women of childbearing age, and alkylating agents like cyclophosphamide (CTX) and leucovorin have clear reproductive toxicity effects on the female reproductive system, resulting in diminished ovarian reserve function. At present, there are no effective means for preventing and treating POI, and there are also concerns regarding protecting the fertility of young women exposed to gonadotoxic chemotherapy (Eunyoung et al., 2021).

*Angelica sinensis* polysaccharide (ASP) is the main pharmacologically active component of *Angelica sinensis* and has been shown to have important *ex vivo* and *in vivo* pharmacological activities, such as immunomodulation, antioxidant, antitumor, antiradiation, and hepatoprotective properties (Yang et al., 2020). Oxidative-stress-induced apoptosis is the main mechanism by which chemotherapeutic drugs damage the ovaries. Several studies have shown that ASP exhibits antioxidant activity *in vivo* by inhibiting the production of reactive oxygen species (ROS) and modulating several chemicals involved in oxidative stress responses (Chao et al., 2020). In the treatment of mice with immune-related premature ovarian failure, the use of ASP was shown to suppress the oxidative stress level and improve ovarian function (Li et al., 2019). However, the mechanism by which ASP exerts protective effects on chemotherapy-injured POI remains to be studied.

In recent years, several studies have gradually revealed the associations between gut flora and female reproductive health disorders. The intestinal flora are also strongly associated with POI. It has been shown that women with POI have significantly dysbiotic intestinal flora, with significantly reduced *Firmicutes* and significantly increased *Firmicutes* to *Bacteroidota* ratio (F/B) (Jiaman et al., 2021). Chemotherapeutic agents are known to significantly affect intestinal microorganisms; CTX damages the intestinal epithelial barrier and increases gastrointestinal permeability, which could lead to intestinal dysbiosis that may also cause chemotherapeutic-agent-induced ovarian damage and promote the development of POI. Therefore, modulating the intestinal flora can be considered a new strategy for treating POI.

In the present study, we established a rat model of POI induced by combining CTX with leucovorin chemotherapy to validate the protective effects of ASP intervention on the ovarian functions of rats, analyze the effects of the community structure of intestinal flora and differences in bacterial genus, and provide a new theoretical basis for the prevention and treatment of POI using ASP. Currently, nearly all evidence involving treatment using ASP stems from animal studies. However, there is a critical lack of rigorously designed randomized controlled trials (RCTs) to validate the efficacy, safety, optimal dosage, and administration regimen of ASP in patients with POI. Hence, clinical drug trials should be conducted in the future to address these gaps.

## 2 Materials and methods

### 2.1 Materials and reagents

ASP was purchased from Shaanxi Ciyuan Biotechnology Co., Ltd. (batch number DG240324) and had a polysaccharide purity of 98%. Busulfan and CTX were purchased from Ron Biotechnology. Malondialdehyde (MDA) and superoxide dismutase (SOD) detection kits were purchased from Wuhan Savier Biotechnology Co., Ltd. The glutathione peroxidase (GSH-Px) assay kit was purchased from Nanjing Jiancheng Bioengineering Institute. The superoxide anion-derived ROS (DHE) detection kit was purchased from Shanghai Biyuntian Biotechnology Co., Ltd. The 4% paraformaldehyde tissue fixative and phosphate-buffered saline (PBS) buffer were purchased from Guangzhou Yitao Biotechnology Co., Ltd.

### 2.2 Animal study and ethical approval

Forty 8-to-10-week-old specific-pathogen-free (SPF) Sprague–Dawley (SD) female rats of bodyweights 220 ± 20 g were selected and purchased from Changsha Tianqin Biotechnology Co. Ltd. (animal license number: SCXK (Xiang) 2024-0021). These animals were bred in the SPF-grade laboratory animal room of Zunyi Medical University Laboratory Animal Center. The rearing conditions included 12/12 h of daylight/darkness cycles each day, an indoor temperature of 22 ± 2 °C, an indoor relative humidity of 50% ± 10%, and *ad libitum* access to food and water. After 1 week of acclimatization to feeding, animals with bodyweights of 220 ± 20 g were enrolled in the experiments. This study was approved by the Ethics Committee of Zunyi Medical University (approval no. Lun Audit (2023)-2-2-296).

### 2.3 Animal model construction, grouping, and drug administration

After all the rats were acclimatized and fed for 1 week, 40 healthy female rats with normal estrous cycles were randomly chosen and divided into five groups (n = 8 per group) under the control, model, as well as low, medium, and high dose ASP (LASP, MASP, and HASP) categories. Except for the control group, all other rats were administered a single intraperitoneal injection of CTX (83.52 mg/kg) with busulfan (20.88 mg/kg) to establish a combined chemotherapy POI rat model; at this step, the control rats were given an equal amount of physiological saline (normal saline or NS) intraperitoneally. The low-dose group was given a gavage of 80 mg/kg/d of ASP, while the medium-dose group was given a gavage of 160 mg/kg/d and high-dose group was given a gavage of 320 mg/kg/d for 21 d. The control and model groups were given a gavage of equal amounts of NS at this time. The general conditions of the rats were monitored daily, including mental status, hair, diet, urination, and defecation, and their weights were measured once a week.

### 2.4 Sample collection

Fasting and drinking management were implemented 12 h before sample collection. Then, anesthesia was administered as an intraperitoneal injection of 2% pentobarbital sodium at a dose of 0.2 mL/100 g bodyweight. After anesthesia, orbital blood was collected from the eyes into a blood collection tube, rested for 30 min, and centrifuged at 4,000 rpm for 15 min before collecting and storing the upper layer of serum in a refrigerator at −80 °C. After blood collection and euthanasia, the rats were dissected in an upward cascade to remove the bilateral ovaries along the uterus by separating the surrounding tissues with gentle movements to reduce mechanical injuries from pulling and squeezing; the ovarian tissues were thus retrieved and washed with NS to remove the blood stains, and the wet weight was determined immediately once the filter paper absorbed the excess moisture from the surface of the ovaries. The ovarian index was calculated as {ovary weight (mg)/bodyweight (g)} × 100%. One side of the ovarian tissue was then immersed in 4% paraformaldehyde fixation solution and stored at room temperature, while the other side of the ovarian tissue was stored at −80 °C in the refrigerator for subsequent experimental manipulations.

### 2.5 Morphological observation of ovarian tissue and follicle counting

The rats were euthanized 12 h after the drug intervention, and the ovarian tissues were separated and removed for hematoxylin and eosin (H&E) staining. The specimen slides were then prepared as follows: (1) The fresh specimens were fixed with 4% paraformaldehyde, dehydrated with ethanol, completely embedded in paraffin, and cut into sections of 3.0 μm thickness; these sections were complete and flat. (2) The sections were placed in an electrically heated oven at 65 °C until the paraffin dissolved. (3) Next, the sections were immersed in xylene I for 10 min and placed in a baking numbered oven at 65 °C; then, it was again immersed in xylene I for 10 min before being placed in sequentially diluted alcohol (100%, 100%, 95%, 95%, 95%, 85%, and 75%) for 8 min and rinsed with distilled water for 1 min. (4) The sections were stained with hematoxylin for 10 min, washed, and differentiated for 15 s using hydrochloric acid alcohol before reverse bluing in tap water for 10 min and staining with eosin for 1 min. (5) The slides were dried in an electric oven at 65 °C, sealed with a neutral resin, observed microscopically and photographed, and the numbers of follicles at all levels (including primordial, primary, secondary, mature, and atretic follicles) were recorded for each group.

### 2.6 ELISA for serum sex hormone and proinflammatory cytokine levels

The serum samples were analyzed using a commercial ELISA kit (Shanghai Jianglai Bio, JL-11525, JL-11706, JL-13251, JL-12462, JL-20896, JL-20884, and JL-13202) according to manufacturer instructions. The procedures included preparing the sample layout table, reconstituting the reagent standards, washing the microplate wells, adding the samples and reagents, incubating the samples, washing the plates, and finally adding the chromogenic substrate followed by the stopping solution. Immediately after processing, the absorbance (A) of each well was measured at 450 nm using a microplate reader. A standard curve was generated and used to calculate the serum levels of estradiol (E2), luteinizing hormone (LH), FSH, and anti-Müllerian hormone (AMH). Furthermore, the serum levels of interleukin-6 (IL-6), interleukin-1β (IL-1β), and tumor necrosis factor-α (TNF-α) were measured using the same method.

### 2.7 Determination of oxidative stress levels in ovarian tissue

Fresh rat ovary tissue (1 g) was retrieved from each group, to which we added saline (9 mL) before placing on ice for homogenization; each sample was then centrifuged, supernatant was removed, and changes in the SOD, MDA, and GSH-Px levels of the ovarian tissue were detected using biochemical kits.

### 2.8 Detection of ROS in ovarian tissue by DHE fluorescent probe

Here, the steps used for tissue paraffin embedding and sectioning were identical to those used for the morphological observations. To stain the nucleus, a circle was drawn around the tissue to prevent the liquid from flowing away, and the tissue was incubated with DHE diluted with PBS at 37 °C for 30 min under dark conditions. For the nuclear staining, the slides were washed in PBS (pH 7.4) on a decolorizing shaker for 5 min each time, and the slides were dried slightly before staining with DAPI solution for 10 min at room temperature away from light. To seal the sections, the slides were placed in PBS (pH 7.4) on a decolorizing shaker and washed thrice for 5 min each with shaking while avoiding light. The slices were then slightly shaken dry before being sealed with an antifluorescence quenching sealer.

### 2.9 16S rDNA sequencing to detect intestinal flora changes

Fecal samples were collected from the rats before euthanasia and stored at −80 °C. Then, DNA was extracted and the V3–V4 region of the bacterial 16S rDNA gene was amplified by polymerase chain reaction. The amplified products were sequenced on the Illumina MiSeq platform (Shanghai Meiji) according to standard protocols, and sequences with ≥97% similarity were clustered into operational taxonomic units (OTUs). Following taxonomic analyses of representative OTU sequences, we assessed the microbiota diversity, inter-sample differences, and gut microbial community composition.

### 2.10 Statistical analysis

SPSS 29.0 software was used for statistical data analyses, and the measured data were expressed as mean ± standard deviation (±s). One-way ANOVA was used for multiple samples, the least significant difference t-test was used for pair comparisons if the variance was uniform, and Tamhane’s T2 test was used for pair comparisons if the variance was non-uniform. Multiple comparisons were performed using repeated-measures ANOVA, and the differences were considered to be statistically significant when *p* < 0.05.

## 3 Results

### 3.1 Therapeutic effects of ASP on POI rats

#### 3.1.1 Effects of ASP on bodyweight and ovarian index in POI rats

**FIGURE 1:**
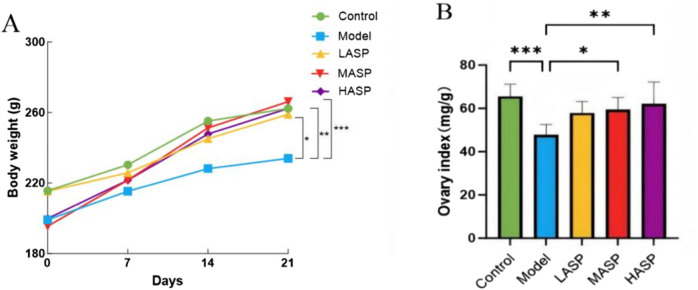
Effects of *Angelica sinensis* polysaccharide (ASP) on the **(A)** bodyweights and **(B)** ovarian index values of rats with premature ovarian insufficiency (POI). The prefixes L, M, and H in the ASP groups refer to low, medium, and high intervention dosages. *p < 0.05, **p < 0.01, ***p < 0.001.

The changes in the bodyweights of the rats (measured once a week) are shown in Figure 1A. The bodyweights of the rats increased gradually with increase in treatment administration duration. However, the bodyweights of rats in the model group increased slowly compared to those in the control and ASP intervention groups. By day 21, the bodyweight differences of the control and ASP intervention groups were statistically significant compared to those of the model group (*p* < 0.001). The ovarian index results were observed to be significantly lower in the model group than the control group (*p* < 0.001). In addition, the ovarian index values were elevated in the ASP intervention groups compared to the model group, with no significant difference compared to the LASP group but statistically significant differences compared to the MASP and HASP groups (*p* < 0.05 and *p* < 0.01, respectively), as shown in Figure 1B.

#### 3.1.2 Effects of ASP on ovarian tissue morphology and follicles in POI rats

**FIGURE 2:**
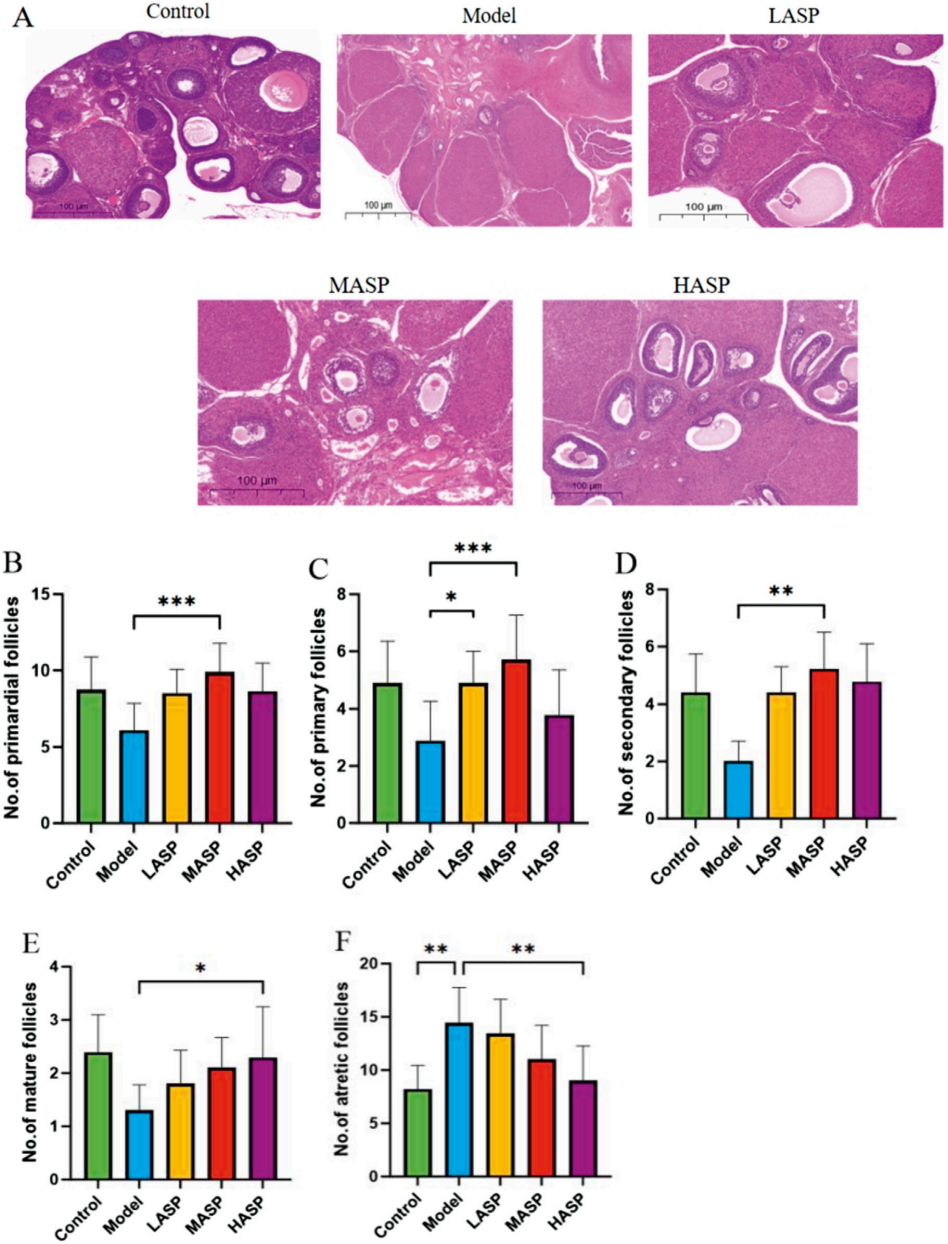
Hematoxylin and eosin (H&E) staining along with follicle counts in the rat ovaries in each group. **(A)** Representative images of H&E staining of rat ovarian tissue in each group (scale bar = 100 μm); counts of the **(B)** primordial; **(C)** primary; **(D)** secondary; **(E)** mature; **(F)** atretic follicles in the experimental groups. *p < 0.05; **p < 0.01.

In the control group, the follicles were structured at all levels, and the granulosa cells were arranged neatly and closely. In the model group, there were fewer follicles in the ovaries, and the granulosa cells were arranged irregularly. In the ASP intervention groups, the numbers of follicles were significantly increased at all levels, and the granulosa cells were arranged more densely compared to the model group (Figure 2A). Upon follicle counting, the model group showed fewer follicle growth at all levels than the control group; although this decrease was not statistically significant, the atretic follicles showed a significant increase (*p* < 0.01), indicating an initial success with the POI rat model. Compared to the model group, the low-, medium-, and high-dose ASP groups showed increased follicle growths at all levels. Here, compared to the model group, the primordial, primary, and secondary follicles increased significantly (*p* < 0.01) in the MASP group; mature follicles increased (*p* < 0.05) and atretic follicles reduced (*p* < 0.01) significantly in the HASP group; and the increases in follicles at all levels were not statistically significant in the LASP group, as shown in Figures 2B–F.

#### 3.1.3 Effects of ASP on serum hormone levels in POI rats

**FIGURE 3:**
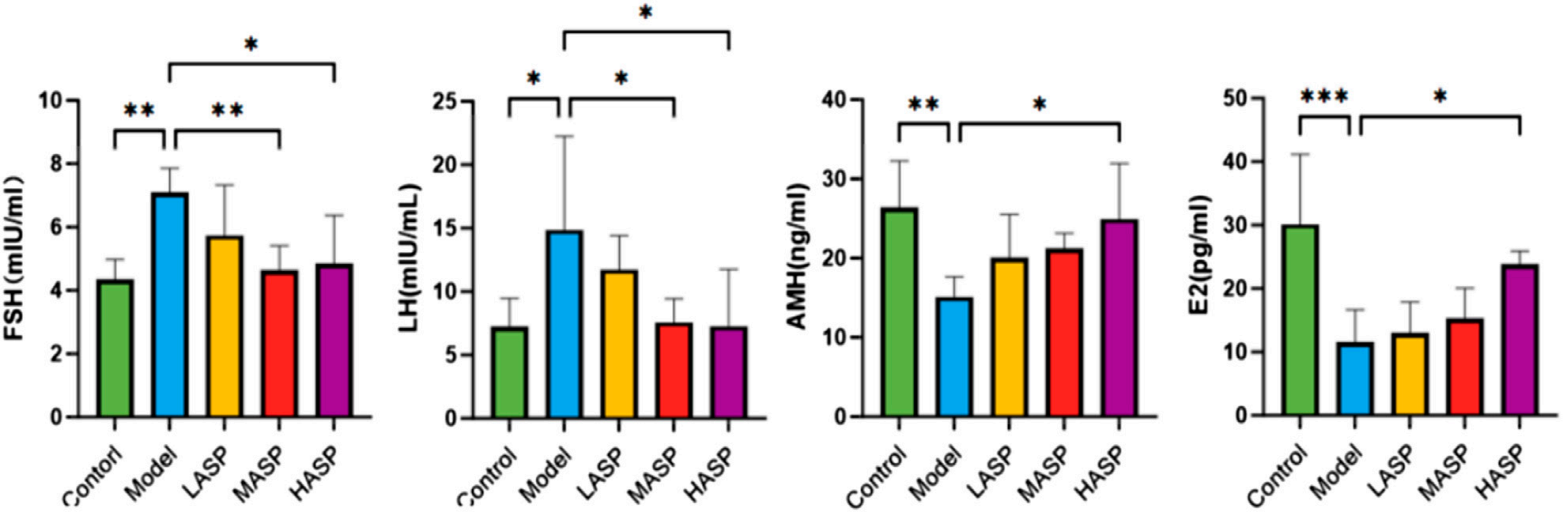
Serum levels of follicle-stimulating hormone (FSH), luteinizing hormone (LH), anti-Müllerian hormone (AMH), and estradiol (E2) in the different groups of rats in this study. *p < 0.05, **p < 0.01, and ***p < 0.001.

The serum sex hormone levels are important for assessing ovarian function, and the results found in this study are shown in Figure 3. Compared to the control group, the serum FSH and LH levels were significantly higher in the model group rats (FSH: *p* < 0.01, LH: *p* < 0.05), while the E2 and AMH levels were significantly lower (E2: *p* < 0.001, AMH: *p* < 0.01). Compared to the model group, FSH and LH levels in the ASP groups decreased by different degrees, and the differences in the FSH and LH levels between the medium- and high-dose groups were statistically significant; E2 levels gradually increased with increase in ASP dose, and the increase in E2 level in the high-dose group was statistically significant (*p* < 0.05); AMH levels increased by different degrees in the different ASP intervention groups, and the increase in AMH level in the high-dose group was statistically significant (*p* < 0.05). Combining the H&E staining results of the ovarian tissue with the serum hormone assay findings, we note that the POI modeling was successful in the rats, the ovarian functions were significantly impaired after modeling, and there were significant improvements in the ovarian function after treatment with ASP.

### 3.2 Effects of ASP on serum proinflammatory cytokine levels in POI rats

**FIGURE 4:**
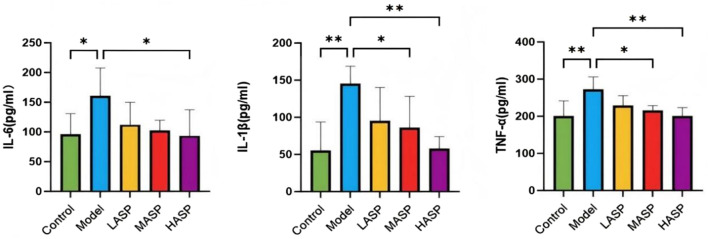
Serum levels of proinflammatory cytokines (IL-6, IL-1β, and TNF-α) in the different groups of rats in this study. *p < 0.05; **p < 0.01.

Compared to the control group, the serum levels of IL-6, IL-1β, and TNF-α were statistically significantly elevated in the model group. Compared to the model group, the individual proinflammatory cytokines were dose-dependently reduced in the different drug intervention groups, with no statistically significant differences noted in the ASP low-dose group; however, there were notable differences in the ASP medium-dose (IL-1β: *p* < 0.05, TNF-α: *p* < 0.05) and high-dose (IL-1β: *p* < 0.01, TNF-α: *p* < 0.01) groups. The IL-6 levels were statistically significant in the ASP high-dose group compared to the model group (*p* < 0.05), and the proinflammatory cytokine levels were not statistically significantly different among the different ASP dose groups, as shown in Figure 4.

### 3.3 Effects of ASP on ovarian oxidative stress levels in POI rats

**FIGURE 5:**
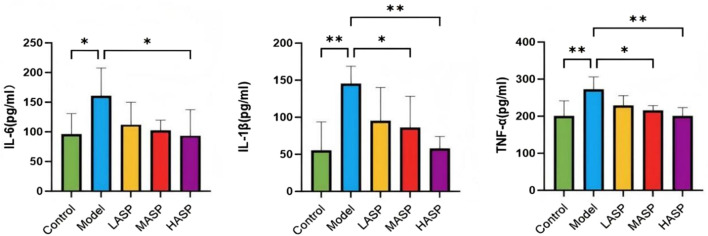
Levels of ovarian oxidative stress indicators superoxide dismutase (SOD), malondialdehyde (MDA), and glutathione peroxidase (GSH-Px) in the different groups of rats in this study. *p < 0.05; **p < 0.01.

The detected levels of oxidative stress markers in the ovarian tissues of rats in different groups are shown in Figure 5. Compared to the control group, the model group exhibited significantly reduced activities of the antioxidant enzymes SOD and GSH-Px (*p* < 0.01), whereas the level of the peroxidation product MDA was elevated. Following ASP administration, the medium-dose group showed significantly increased SOD and GSH-Px activities (*p* < 0.05) and significantly decreased *MDA* level (*p* < 0.01). The high-dose ASP group also showed significantly elevated SOD activity (*p* < 0.05).

**FIGURE 6:**
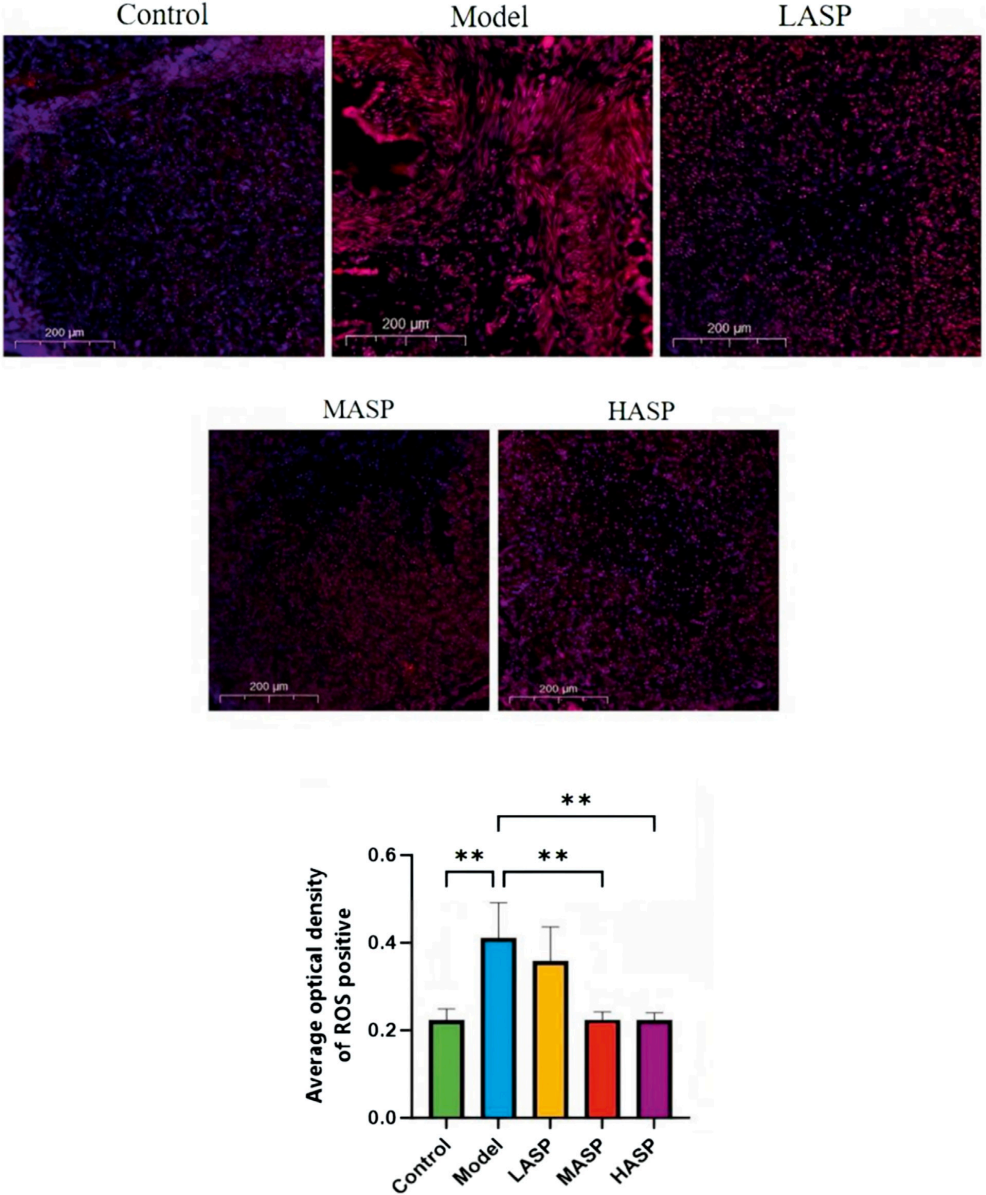
Superoxide anion-derived reactive oxygen species (DHE) fluorescence probe labeling and quantification of reactive oxygen species (ROS) levels in the ovarian tissues of rats in different experimental groups. **p < 0.01, scale bar = 200 μm.

The ROS levels in the ovarian tissues were assessed using the DHE fluorescent probe (Figure 6). Compared to the control group, the model group exhibited extensive DHE-positive staining in the ovarian tissues, indicating significantly elevated ROS levels. However, the DHE-positive areas were markedly reduced across different dosage groups compared to the model group following ASP intervention and showed significant decreases in ROS levels (*p* < 0.01). Collectively, these results demonstrate that ASP intervention significantly alleviates oxidative stress in the ovarian tissues of POI rats, with notable effects observed in the medium- and high-dose groups.

### 3.4 Analysis of differences in intestinal flora

#### 3.4.1 Differences in the overall community structure of rat intestinal flora between groups

**FIGURE 7:**
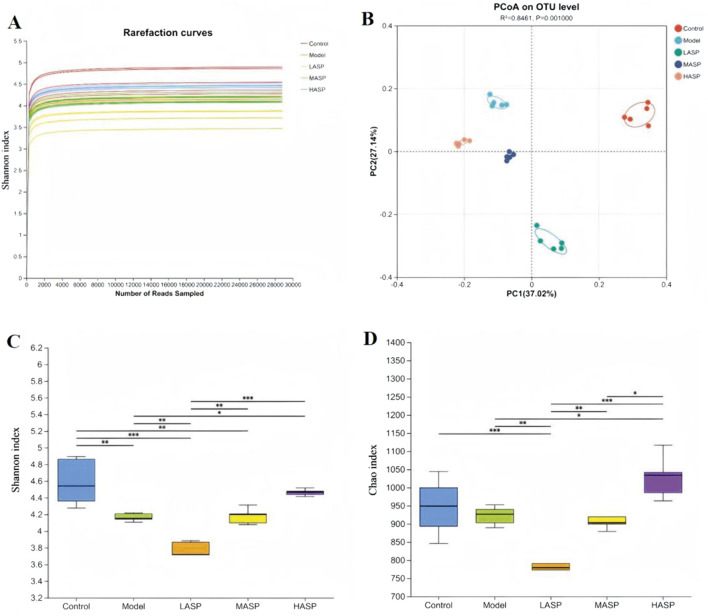
Differences in the overall structure of gut microbiota among the experimental groups. **(A)** Dilution graphs showing evaluation of the reasonability of the sequencing data. **(B)** Principal coordinate analysis (PCoA) plot showing clustering of beta diversity among the groups. **(C)** Shannon and **(D)** Chao indexes showing the alpha diversity among the groups. *p < 0.05, **p < 0.01, ***p < 0.001.

The structural and functional differences in the intestinal flora of each group of rats were analyzed via 16S rDNA sequencing. First, a sparsity curve was used to evaluate the reasonability of the rat intestinal flora sequencing data in each group. The data were observed to stabilize when the number of sequenced samples reached 4,000 in each group (Figure 7A), ensuring that the sequencing data were reliable and reasonable enough to cover all microbial species in the samples. The Shannon and Chao indexes were used to analyze the α-diversity of the intestinal flora, which showed significant differences among the groups (Figures 7C,D); this indicated that chemotherapy and ASP intervention had significant effects on the α-diversity of the intestinal flora. The β-diversity of the intestinal flora among the different groups was calculated using the Bray–Curtis distance, and the principal coordinate analysis (PCoA) was used to produce the results shown in Figure 7B with an Adonis test of *p* = 0.001; these results show a significant tendency for clustering of the β-diversity among the groups with the control group as origin as well as the model and ASP intervention groups to deviate in different directions. The large sample distances between the model and different ASP groups indicate significant differences in the intestinal flora compositions among these groups.

#### 3.4.2 Differences in intestinal flora at the phylum level among different rat groups

**FIGURE 8:**
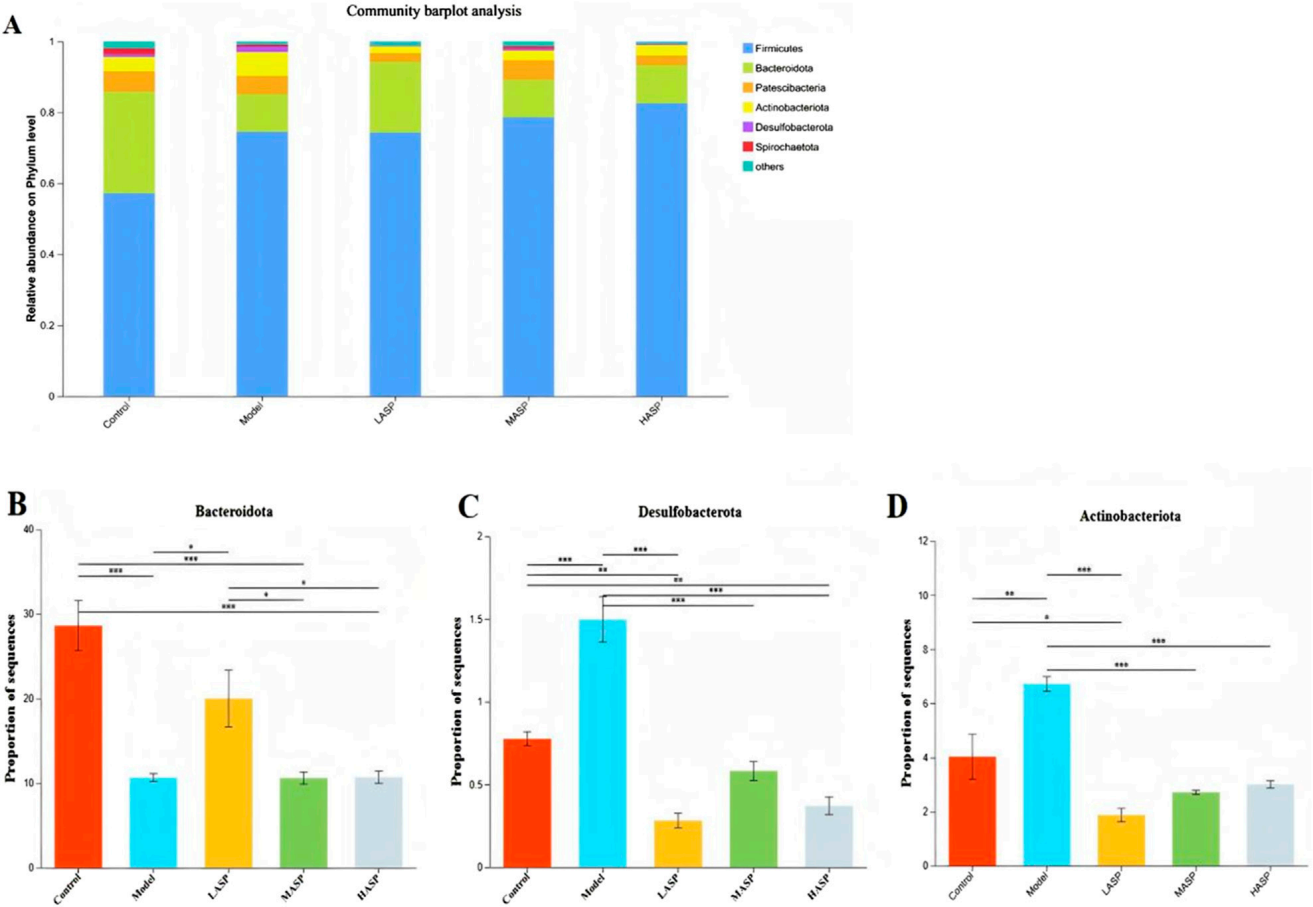
Relative abundances of different bacterial species at the phylum level. **(A)** Distribution of rat intestinal flora at the phylum level. Relative abundances of the phyla **(B)** *Bacteroidota*; **(C)** *Desulfobacterota*; **(D)** *Actinobacteriota* in different experimental groups. *p < 0.05, **p < 0.01, and ***p < 0.001.

Differences in gut microbiota compositions among the experimental groups were first analyzed at the phylum level. A histogram of the results shows the relative abundances of the rat intestinal flora distributed at the phylum level (Figure 8A). Here, *Firmicutes* and *Bacteroidota* dominate the intestinal bacterial community, accounting for approximately 86% of the overall bacterial community structure. Next, *Actinobacteria*, *Patescibacteria*, *Proteobacteria*, *Campylobacteria*, and *Desulfobacteria* account for approximately 10% of the total structure, while other phyla were present with low relative abundances. Compared to the control group, the model group exhibited a lower relative abundance of *Bacteroidota* but higher relative abundances of *Verrucomicrobiota* and *Actinobacteriota*. Following ASP treatment, the relative abundance of *Bacteroidota* increased, while those of *Actinobacteriota*, *Patescibacteria*, and *Desulfobacterota* decreased. These findings demonstrate that ASP intervention can restore chemotherapy-induced dysbiosis of the intestinal flora at the phylum level in rats with POI, and the corresponding results are detailed in Figures 8B–D.

#### 3.4.3 Differences in intestinal flora at the genus level among different rat groups

**FIGURE 9:**
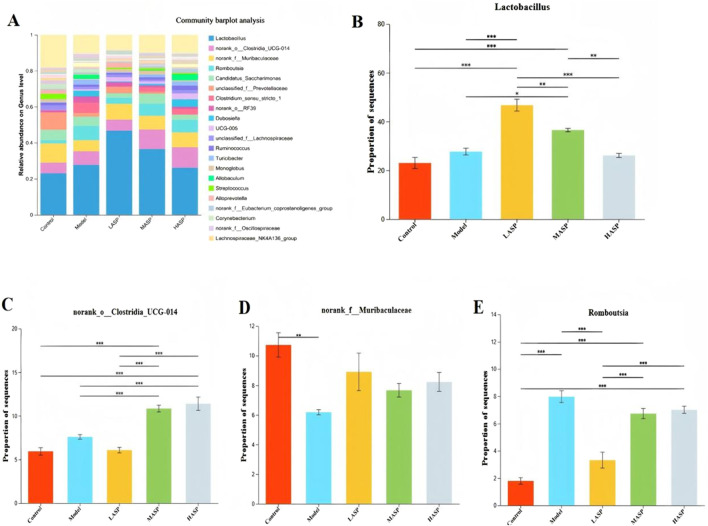
Relative abundances of different bacterial species at the genus level. **(A)** Distribution of rat intestinal flora at the genus level. Relative abundances of the genera **(B)** *Lactobacillus*; **(C)** norank_o_Clostridia_UCG-014; **(D)** norank_f_Muribaculaceae; **(E)** *Romboutsia*. *p < 0.05, **p < 0.01, and ***p < 0.001.

Differences among the gut microbiota of the experimental groups were also analyzed at the genus level of classification. The results showed that *Lactobacillus* (*Lactobacillus*), norank_o_Clostridia_UCG-014, norank_f_Muribaculaceae, and *Romboutsia* were the dominant genera in all groups (Figure 9). Compared to the control group, the relative abundances of norank_f_Muribaculaceae, Candidatus_Saccharimonas, unclassified_f_Prevotellaceae, unclassified_f_Lachnospiraceae, *Ruminococcus* (*Ruminococcus*), *Monoglobus*, and *Streptococcus* decreased in the model group. The ASP intervention groups showed increased relative abundances of *Lactobacillus* spp, norank_o_Clostridia_UCG-014, and norank_f_Muribaculaceae. Thus, we demonstrated that ASP could also improve the intestinal flora at the genus level.

#### 3.4.4 Correlation analysis between differential flora and related indicators

**FIGURE 10:**
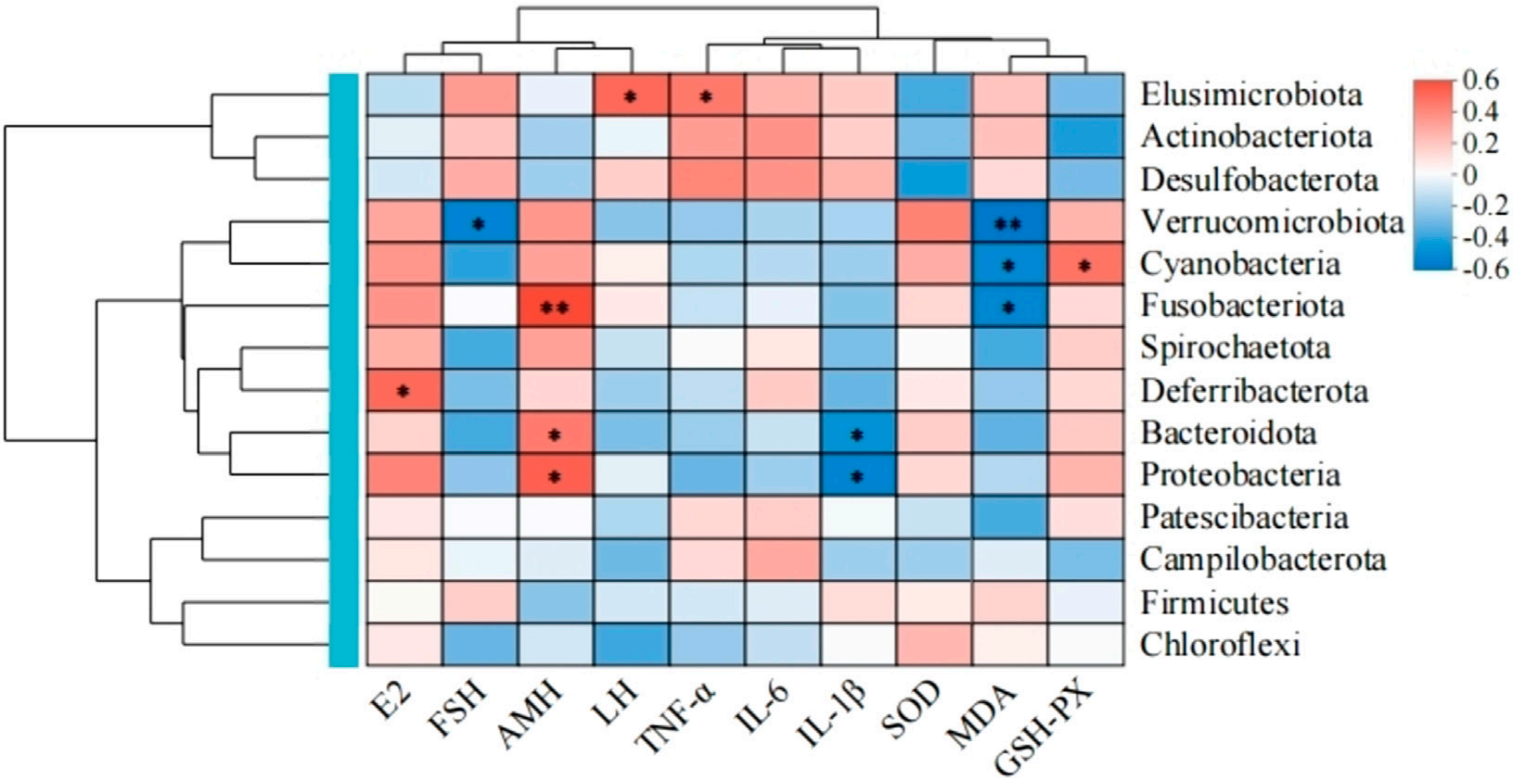
Spearman’s correlation analysis of the gut microbiota in the experimental rats and their related indicators.

Spearman’s correlation analysis was performed between the various species and their relevant indicators based on the key species differences between the groups (Figure 10). The results show that at the phylum level, *Verrucomicrobiota*, *Fusobacteriota*, *Deferribacterota*, *Bacteroidota*, and *Proteobacteria* were positively correlated with serum E2 and AMH levels as well as negatively correlated with FSH and LH levels. The Campylobacterota showed a positive correlation with serum E2 level as well as negative correlations with AMH, FSH, and LH levels; *Actinobacteria*, *Desulfobacteria*, and *Firmicutes* were positively correlated with serum FSH level and negatively correlated with E2 and AMH levels. In terms of oxidative stress homeostasis, the ovarian tissue MDA level was positively correlated with the abundances of phyla *Actinobacteria*, *Desulfovibrio*, and thick-walled *Bacillus* while being negatively correlated with the abundances of phyla *Micrococcus wartyi*, *Clostridium*, *Desulfovibrio*, *Anaplastomycetes*, *Ascomycetes*, and *Campylobacter*. The antioxidant enzyme correlation analysis showed contrasting results, with the SOD and GSH-Px activities positively correlated with the abundances of phyla *Micrococcus wartyi*, *Clostridium*, *Desmodium*, *Anaplasma*, and *Ascomycetes* but negatively correlated with the abundances of phyla *Actinobacteria*, *Desmodium*, and *Campylobacter*. In the correlation analysis with proinflammatory cytokines, the serum IL-6, IL-1β, and TNF-α levels were positively correlated with the abundances of phyla *Actinobacteria* and *Desulfovibrio* but negatively correlated with the abundances of phyla *Micrococcus wartimei*, *Clostridium* sp., *Mycobacterium anomalum*, and *Aspergillus*. In addition, the serum IL-6 level was positively correlated with the abundances of phyla *Desulfovibrio* and *Campylobacter* but negatively correlated with the abundances of thick-walled phylum. The serum TNF-α level was positively correlated with the abundance of phylum *Campylobacter* but negatively correlated with the abundances of *Desmodium* and thick-walled phyla. These findings indicate that there are close correlations and interactions among the above intestinal flora and serum sex hormone, proinflammatory cytokine, and oxidative stress levels in ovarian tissues.

## 4 Discussion

In recent years, the number of young women suffering from malignant tumors has increased gradually. Given the widespread use of chemotherapeutic drugs, the long-term survival rates of tumor patients have increased gradually; however, these drugs also seriously affect the physical, psychological, and reproductive health of such young women (Guihai et al., 2023). Chemotherapeutic drugs inevitably cause damage to normal cells, leading to DNA damage and oxidative stress in both somatic and germ cells. In most cases, there is severe damage to the intestinal mucosa, which may further cause intestinal-related problems such as bacterial translocation, metabolic disorders, and imbalance of intestinal flora (Yang et al., 2013; Tong et al., 2020). Studies have shown that chemotherapeutic drugs may have long-term toxicity and harmful effects on the oocytes and ovaries, which could lead to female reproductive dysfunction as well as irreversible ovarian failure; these could cause loss of primordial follicles and ovarian atrophy, contributing to apoptosis of the granulosa cells and affecting the stability of the ovarian microenvironment, leading to temporary amenorrhea or even development of premature ovarian failure (POF), thereby reducing fertility in young women.

Chemotherapy can also induce direct ovarian damage by causing DNA double-strand breaks (DSBs). Alternatively, it can act indirectly by increasing oxidative stress or disrupting the ovarian microvasculature, leading to localized ischemia, necrosis, or even inflammation that ultimately contribute to primordial follicular depletion (Kashi and Meirow, 2023). Chemotherapy can also exacerbate ovarian damage by impairing the ovarian microenvironment; this typically manifests as abnormal extracellular matrix deposition, interstitial fibrosis, angiogenic dysregulation, immune microenvironment imbalance, oxidative stress imbalance, and ovarian stem cell depletion, thereby compromising ovarian function further (Guo et al., 2024). Currently, chemotherapy models are the most established method for POI modeling, with the CTX-induced model being the most representative in terms of current research (Juan et al., 2022).

In this study, we established chemotherapy-induced ovarian injury in the POI rat model via intraperitoneal injection of CTX along with busulfan. Following modeling, the rats exhibited various symptoms like thinning and dullness of fur, reduced food intake, slow weight gain, and occasional loose stools. Our experimental results demonstrated that compared to the control group, the model group showed significant reduction in ovarian weight, elevated serum levels of FSH and LH, and markedly decreased levels of E2 and AMH. H&E staining of the ovarian tissue revealed ovarian atrophy, irregular follicular morphology, significant reductions in follicles at all developmental stages, and increased number of atretic follicles. Collectively, these findings indicate significant impairment of ovarian function in the rats, confirming successful establishment of the POI model.

Traditional Chinese medicine (TCM) is a systematic discipline crystallized from millennia of medical practice in China and offers distinct advantages in treating patients with ovarian insufficiency, including a favorable safety profile, significant efficacy, and convenient usage. Thus, the development and application of classical TCM formulas are of great significance for innovative inheritance of this tradition. *Angelica sinensis* (Danggui) is an *Umbelliferae* plant and a core medicinal herb within the TCM system; it has been documented for therapeutic use since Shennong’s Classic of the Materia Medica and is renowned as the “sacred herb of the blood.” The Gansu province of China is a major producer of high-quality *A. sinensis*, which is characterized by high yield and superior efficacy. Modern pharmacological research efforts have confirmed that the warming properties of *A. sinensis* (which enters the liver, spleen, and heart meridians) are closely associated with its multitarget regulatory effects. These effects encompass tonifying and invigorating blood, regulating menstruation, alleviating pain, and moistening the intestines to promote laxation. Therefore, based on TCM theories, *A. sinensis* and its active ingredients demonstrate significant therapeutic potential for POI. *A. sinensis* is rich in polysaccharides, which are key bioactive components with diverse pharmacological effects, including antioxidant, anti-inflammatory, immunomodulatory, intestinal protective, hypoglycemic, and neuroprotective activities (Jijuan et al., 2021). Research has shown that ASP can alleviate inflammation and oxidative stress by downregulating the expressions of cellular inflammatory factors and related genes, while upregulating antioxidant enzyme genes and proteins. Notably, ASPs from different structural parts of *A. sinensis* exhibit varying anti-inflammatory and antioxidant activities (Yuan-Feng et al., 2022). Furthermore, herbal polysaccharides, including ASPs, are reported to regulate intestinal functions through three interconnected pathways, namely, modulating intestinal immunity, reinforcing the intestinal barrier, and balancing intestinal flora, which collectively contribute to maintaining overall health.

In this study, we investigated three dosing levels of ASP, namely, low, medium, and high, as intervention in rats with chemotherapeutic injury in the POI model. After treatment, the ovary weights increased significantly, and the difference between the medium- and high-dose ASP groups was statistically significant. H&E staining of the ovarian tissues showed that the tissue structure improved significantly after ASP intervention, with the granulosa cells being arranged in neat rows, increased numbers of follicles at all growth levels, and reduced atretic follicles, suggesting that ASP has potential therapeutic benefits in improving ovarian tissue morphology, promoting follicular development, and preventing follicular atresia. The ELISA test for serum sex hormone levels in rats showed that compared with the model group, the serum E2 and AMH levels in the ASP intervention groups increased significantly, while the FSH and LH levels decreased significantly; here, the decreases in FSH and LH levels in the medium- and high-dose groups as well as increases in E2 and AMH levels in the high-dose group were statistically significant. E2 plays a pivotal role in follicular development and is primarily secreted by the follicular cells, underscoring its close association with ovarian function (Ueno et al., 2025). Conversely, AMH is a transforming growth factor-β (TGF-β) glycoprotein hormone secreted by the granulosa cells and serves as the most reliable biomarker for assessing ovarian reserve as well as predictor of ovarian response to ovulation induction therapy (Akira et al., 2023). Consequently, AMH is a more sensitive diagnostic indicator for the early clinical detection of POI. LH and FSH are both gonadotropins; here, FSH is the key driver of early follicular growth, development, and estradiol synthesis, while LH activity is critical for folliculogenesis and luteinization that collaborate with FSH to ensure physiological progression from dominant follicle development through ovulation to luteinization (Ginther, 2022). The above findings indicate that ASP can significantly improve ovarian function, with the medium- and high-dose intervention groups showing the most remarkable therapeutic effects.

The ovarian toxicity of CTX likely stems from its induction of oxidative stress, inflammation, and other detrimental responses within the ovarian tissues. Oxidative stress is cause by excess production of ROS as well as imbalance between the oxidative and antioxidant systems, leading to cellular damage (Lijuan et al., 2016); it is considered a key factor in initiating or promoting female reproductive disorders (Wang et al., 2025). In the ovary, excessive ROS levels can trigger heightened apoptosis of the granulosa cells and oocytes, contributing to POI (Hayes and Dinkova-Kostova, 2014). Further, chemotherapy-induced chronic inflammation causes progressive tissue damage in the ovary accompanied by alterations in the inflammatory and immune cell phenotypes, ultimately resulting in diminished ovarian reserve (Liu et al., 2019). Notably, POI is frequently associated with elevated systemic and local inflammatory markers. In this study, ELISA of the rat serum revealed significantly elevated levels of the proinflammatory cytokines IL-6, IL-1β, and TNF-α in the model group compared to the control group. Intervention with varying doses of ASP reduced these inflammatory factor levels in a dose-dependent manner. Concurrently, ovarian tissue analysis demonstrated significant increases in ROS and the lipid peroxidation product MDA, alongside significant decreases in the antioxidant enzymes GSH-Px and SOD in the model group, indicating severe oxidative damage. Compared to the model group, the ASP treatment groups exhibited increased levels of GSH-Px and SOD, along with decreased MDA and ROS levels in the ovarian tissues. These findings collectively suggest that ASP protects ovarian tissues from inflammatory and oxidative stress damage by enhancing the anti-inflammatory and antioxidant capacities of the body, thereby improving ovarian function.

Intestinal flora are known to significantly influence chemotherapy outcomes by modulating both the antitumor efficacies and toxicities of chemotherapeutic agents, positioning them as a key concern in individualized cancer treatment in the future. Chronic inflammation resulting from intestinal dysbiosis has been shown to impair ovarian function; a related study reported reductions in the primordial and antral follicles, an increase in atretic follicles, and a significant decline in ovarian reserve (Ahmed et al., 2024). CTX treatment commonly induces gut dysbiosis, characterized by reduced microbial community richness, proliferation of harmful bacteria, and depletion of beneficial bacteria. To investigate whether similar gut dysbiosis exists in the POI rat model used in this study and to determine if the improvements observed with ASP are associated with gut flora remodeling, we analyzed the gut microbiota composition using 16S rDNA sequencing. Comparison of the α-diversity confirmed that there were significant differences in the intestinal flora composition in the POI rats compared to the control group. Compared to the control group, the Chao and Shannon indexes were lower in the model group, indicating that the microbiological composition of the intestinal flora was less rich and homogeneous. The β-diversity comparisons demonstrated that rats in the POI model group tended to deviate from the control and ASP intervention groups in different directions, which indicates that the diversity of the intestinal flora of the model group is significantly dysfunctional after modeling. In this case, increasing the dose of ASP is expected to gradually improve the dysbiosis of intestinal flora.

Further analysis showed that at the phylum level, the intestinal flora of rats in all groups was dominated by *Firmicutes*, *Bacteroidota*, *Proteobacteria*, and *Actinobacteria*. The thick-walled bacteria and *Mycobacterium anisopliae* accounted for about 90% of the intestinal bacteria *in vivo*. This result is consistent with the findings of previous studies. The thick-walled *Bacillus* and *Bacillus anthropophilus* are two of the most important species in the human gut and play critical roles in maintaining intestinal homeostasis, promoting nutrient absorption, and regulating immunity (Sun et al., 2024; Krawczyk et al., 2025; Jingyi et al., 2022; Zhang et al., 2024). The chemotherapy-induced POI model rats had a relatively low abundance of intestinal *Anaplasma* as well as significantly increased abundances of *Actinobacteria* and *Desulfobacterota*. Treatment with ASP increased the abundance of *Anabaena* and significantly decreased the relative abundances of *Actinobacteria* and *Desulfovibrio*, suggesting that ASP significantly affects the gut microbial community structure. Furthermore, *Bacteroidetes* metabolize host-indigestible dietary fibers to produce short-chain fatty acids that serve as energy sources for intestinal cells, enhance intestinal barrier function, and exert anti-inflammatory effects; these properties are closely linked to conditions like obesity, insulin resistance, dyslipidemia, and inflammation ^[26]^. Significant differences in the gut microbiota compositions at the genus level were also observed among the rat groups; ASP intervention significantly increased the relative abundances of *Lactobacillus*, norank_f_Muribaculaceae, and unclassified_f_Prevotellaceae compared to the model group. *Lactobacillus* belongs to the phylum *Firmicutes*, while the other two genera belong to *Bacteroidota*. These bacteria are essential for maintaining intestinal homeostasis, promoting energy metabolism, and enhancing nutrient absorption. Notably, *Lactobacillus* represents a core group of probiotics that have significant roles in reproductive system protection, including maintaining the vaginal microbiota balance, reducing the risk of preterm labor and intrauterine infections, and conferring multiple host benefits. Although ASP did not modulate these specific microbial changes in a strictly dose-dependent manner, the observed alterations suggest a potential link between the therapeutic effects of ASP and their impacts on these taxa. Furthermore, the relative abundances of the dominant phyla like *Bacteroidota* and *Firmicutes* in the low-dose ASP group closely resemble those of the control group. Given that higher ASP doses appeared to exert an inhibitory effect on the gut flora, subsequent studies investigating gut microbiome modulation using ASP could consider employing lower concentrations and/or prolonged interventions to potentially optimize the therapeutic outcomes.

To evaluate potential relationships among serum sex hormone levels, ovarian tissue inflammation/oxidative stress markers, and gut microbes, we performed Spearman’s correlation analysis; this revealed positive correlations of beneficial bacteria like *Verrucomicrobiota* and *Bacteroidota* with serum E2 and AMH levels. Conversely, *Actinobacteria* and *Firmicutes* showed positive correlations with serum FSH level but negative correlations with E2 and AMH levels. *Verrucomicrobiota* is a key species in maintaining intestinal health and plays vital metabolic, immunologic, and protective roles at the intestinal mucosal barrier. The observed POI-induced reductions in serum E2 and AMH levels along with increase in FSH level may be linked to alterations in the composition of the gut microbiome. Furthermore, the beneficial bacteria appear to have the capability to modulate serum hormone levels. Notably, the relative abundance of *Verrucomicrobiota* was positively correlated with SOD and GSH-Px levels in ovarian tissues and negatively correlated with MDA level, suggesting a potential link between the gut microbiota and antioxidant defense mechanisms. We also observed that the abundance of *Desulfobacterota* was positively correlated with serum levels of the proinflammatory cytokines IL-6, IL-1β, and TNF-α as well as negatively correlated with E2 level. *Desulfobacterota* contributes to intestinal and systemic inflammation primarily through hydrogen sulfide production and immune regulatory imbalance; its elevated abundance can be considered a potential biomarker for inflammatory diseases ^[27]^.

In conclusion, the present study demonstrates that ASP can ameliorate bodyweight loss, restore the ovarian index, and correct hormonal imbalances (elevated E2 and AMH; reduced FSH and LH) in chemotherapy-induced POI rats. ASP also suppresses proinflammatory cytokines (IL-6, IL-1β, and TNF-α) and oxidative stress markers (ROS and MDA), while enhancing the activities of antioxidant enzymes (SOD and GSH-Px). Notably, the efficacy of ASP was more pronounced in the medium- and high-dose groups, and ASP improved the intestinal flora composition. Our preliminary findings show that the protective effects of ASP may possibly be exerted through anti-inflammatory, antioxidant, and gut microbiota remodeling mechanisms; these help us further understand the pathogenesis of POI and provide new experimental evidence for the clinical application of ASP in treating POI.

## 5 Conclusion

ASP can improve the serum sex hormone levels in rats with chemotherapy-induced POI, increase the numbers of follicles at various growth stages, as well as inhibit inflammatory responses and oxidative stress levels, thereby protecting ovarian function. The medium- and high-dose ASP groups in this study show significant therapeutic effects, and ASP can reshape the gut microbiota structure in POI rats. The main differential microbiota are closely related to the serum sex hormone, inflammation, and oxidative stress levels in the ovarian tissues, suggesting that the gut microbiota may provide a new approach for the treatment of POI.

## 6 Limitations

The preliminary findings of the present study indicate that ASP improves chemotherapy-induced POI through its anti-inflammatory, antioxidant, and gut-microbiota-modulating effects; hence, we explored the effective dosage for ASP application. However, the following limitations remain. The mediating roles of key signaling pathways as well as direct interaction patterns between the gut microbiota metabolites and ovaries have not been elucidated at the mechanistic level. Clinical translation of the findings of this study lacks sufficient dose-response and safety data pertaining to humans. The causal relationship between microbiota alterations and ovarian repair requires further validation through fecal microbiota transplantation (FMT). Thus, further investigations at the mechanistic level in future studies could involve examining the relevant signaling pathways, designing FMT experiments, performing fecal metabolite analyses, and conducting more in-depth research on the relationships between the gut microbiota and POI. These would provide a more robust theoretical basis for the clinical treatment of POI.

## Data Availability

The data presented in the study are deposited in the NCBI repository, accession number PRJNA1358839.
